# Increased resistance to proteasome inhibitors in multiple myeloma mediated by cIAP2 - implications for a combinatorial treatment

**DOI:** 10.18632/oncotarget.4139

**Published:** 2015-05-14

**Authors:** Charlotte Fristedt Duvefelt, Susanne Lub, Prasoon Agarwal, Linda Arngården, Anna Hammarberg, Ken Maes, Els Van Valckenborgh, Karin Vanderkerken, Helena Jernberg Wiklund

**Affiliations:** ^1^ Science for Life Laboratory, Department of Immunology, Genetics and Pathology, Rudbeck Laboratory, Uppsala University, Uppsala Sweden; ^2^ Department of Hematology and Immunology, Myeloma Center Brussels, Vrije Universiteit Brussel, (VUB) Brussels, Belgium

**Keywords:** multiple myeloma, cellular inhibitor of apoptosis protein 2, proteasome inhibitors, drug resistance, antagonist of inhibitors of apoptosis proteins

## Abstract

Despite the introduction of new treatment options for multiple myeloma (MM), a majority of patients relapse due to the development of resistance. Unraveling new mechanisms underlying resistance could lead to identification of possible targets for combinatorial treatment. Using TRAF3 deleted/mutated MM cell lines, we evaluated the role of the cellular inhibitor of apoptosis 2 (cIAP2) in drug resistance and uncovered the plausible mechanisms underlying this resistance and possible strategies to overcome this by combinatorial treatment. In MM, cIAP2 is part of the gene signature of aberrant NF-κB signaling and is heterogeneously expressed amongst MM patients. In cIAP2 overexpressing cells a decreased sensitivity to the proteasome inhibitors bortezomib, MG132 and carfilzomib was observed. Gene expression analysis revealed that 440 genes were differentially expressed due to cIAP2 overexpression. Importantly, the data imply that cIAPs are rational targets for combinatorial treatment in the population of MM with deleted/mutated TRAF3. Indeed, we found that treatment with the IAP inhibitor AT-406 enhanced the anti-MM effect of bortezomib in the investigated cell lines. Taken together, our results show that cIAP2 is an important factor mediating bortezomib resistance in MM cells harboring TRAF3 deletion/mutation and therefore should be considered as a target for combinatorial treatment.

## INTRODUCTION

Multiple myeloma (MM) is a post-germinal center B-cell tumor characterized by an accumulation of malignant monoclonal plasma cells in the bone marrow. It is the second most prevalent hematological malignancy and accounts for approximately 1% of all cancers. The increased understanding of the biology of MM has led to the current improvements of treatment, such as the proteasome inhibitor bortezomib, prolonging survival greatly for MM patients [1-5] Thus, novel insights on possible underlying mechanisms for resistance are highly likely to benefit the development of yet novel and improved therapies to MM. A possible explanation for the failure to cure MM is the emergence of drug resistant clones. Different mechanisms have been suggested to lead to bortezomib resistance e.g. mutations and overexpression of the proteasome subunit PSMB5 [6], alterations in genes associated with stress response such as heat shock proteins [7], and up-regulation of cell survival pathways such as the IGF-1/IGF-1R axis [8].

Important mechanisms underlying insensitivity to therapy are genetic alterations in major key signaling pathways. In MM, a constitutive NF-κB activation, as demonstrated by an activated gene signature promotes tumor cell survival and proliferation [9-11]. cIAP2, a member of the inhibitor of apoptosis proteins (IAP) is part of this gene signature. IAPs are frequently found altered in human malignancies, including hematological tumors, leading to aberrant apoptosis-signaling pathways. These alterations are often associated with chemoresistance, disease progression and poor patient prognosis [12-14]. Eight human homologues of IAPs have so far been identified and all contain one to three baculovirus IAP repeat (BIR) motifs [15] that allow them to bind to and inhibit caspases. XIAP (X-chromosome linked IAP) is best characterized and a prominent inhibitor of caspase activation [14, 16]. However, not all homologues of the IAP family have been convincingly shown to exert the anti-apoptotic effect by direct binding and sequestering of caspase activity [17].

The IAP homologues are also important regulators of the NF-κB pathway. The NF-κB transcription factor family consist of five subunits (p50/NF-κB1, p52/NF-κB2, p65, c-Rel and RelB) which dimerize into hetero- and homo-dimeric complexes and are held in the cytoplasm by the inhibitors of NF-κBs (IκBs) [18]. There are two distinct NF-κB activation pathways, the canonical and the non-canonical NF-κB pathway [19, 20]. In the canonical pathway the inhibitor of IκB kinase β (IKKβ) phosphorylates the IκBs leading to proteasomal degradation, and accumulation of p50/p65 and c-rel/p65 in the nucleus [9, 21, 22]. In the non-canonical pathway IKKα is activated by NF-κB inducing kinase (NIK). The activated IKKα phosphorylates NF-κB2 and proteasomal removal of an inhibitory C-terminal IκB-δ domain, resulting in accumulation of p52/RelB in the nucleus [23]. cIAP1/cIAP2 play an important role in both the canonical and non-canonical NF-κB pathway. In the canonical pathway cIAP1/2 are positive regulators of TNFα mediated activation, while in the non-canonical NF-κB pathway, cIAP1/2, TRAF2 and TRAF3 have repressive roles by promoting the ubiquitination and degradation of NIK [14]. Genetic lesions in the non-canonical pathway leading to uncontrolled NF-κB activation appear in approximately 20% of MM patients [9, 14, 24]. Among these, the loss of functional TRAF3 is the most common gene deleted/mutated [9]. Importantly, MM harboring inactivated TRAF3 and a hyperactivated NF-κB signature e.g. high expression of genes that are involved in NF-κB signaling, are associated to initial good response to proteasome inhibitors [24, 25]. In this study we aimed at exploring the role of cIAP2 in emerging drug resistance in MM cells. We focused on the human MM cell lines harboring TRAF3 deletion/mutation (LP-1 and ANBL-6). LP-1 lack the entire MATH domain that is crucial for interaction with NIK and ANBL-6 has a mutation causing production of a protein with a disrupted MATH domain [9].

Since IAPs can block apoptosis-signaling pathways, promote survival and are likely candidates for drug resistance, the IAP proteins are currently considered as promising molecular targets for therapy [26]. In this paper, we have identified an important role of cIAP2 as a regulator of sensitivity of MM cells to proteasome inhibitors in the subgroup with TRAF3 mutation/deletion. Moreover, the IAP inhibitor AT-406 sensitized the MM cells to bortezomib treatment, thereby providing a rationale for a potential therapeutic use of the IAP inhibitor AT-406 in combination with proteasome inhibitors.

## RESULTS

### Overexpressing cIAP2 in TRAF3 deleted/mutated MM cell lines leads to drug tolerance selectively towards proteasome inhibitors

**Figure 1:**
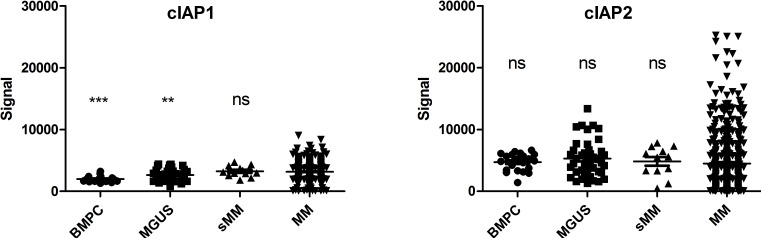
cIAP1 and cIAP2 expression in MM patients cIAP1 and cIAP2 expression in bone marrow plasma cells from healthy donors (*n* = 22), MGUS (*n* = 44), smoldering MM (*n* = 12) and MM patients (n = 414). *** and ** indicate respectively *p* < 0.0001 and *p* < 0.01 compared to MM patients.

**Figure 2:**
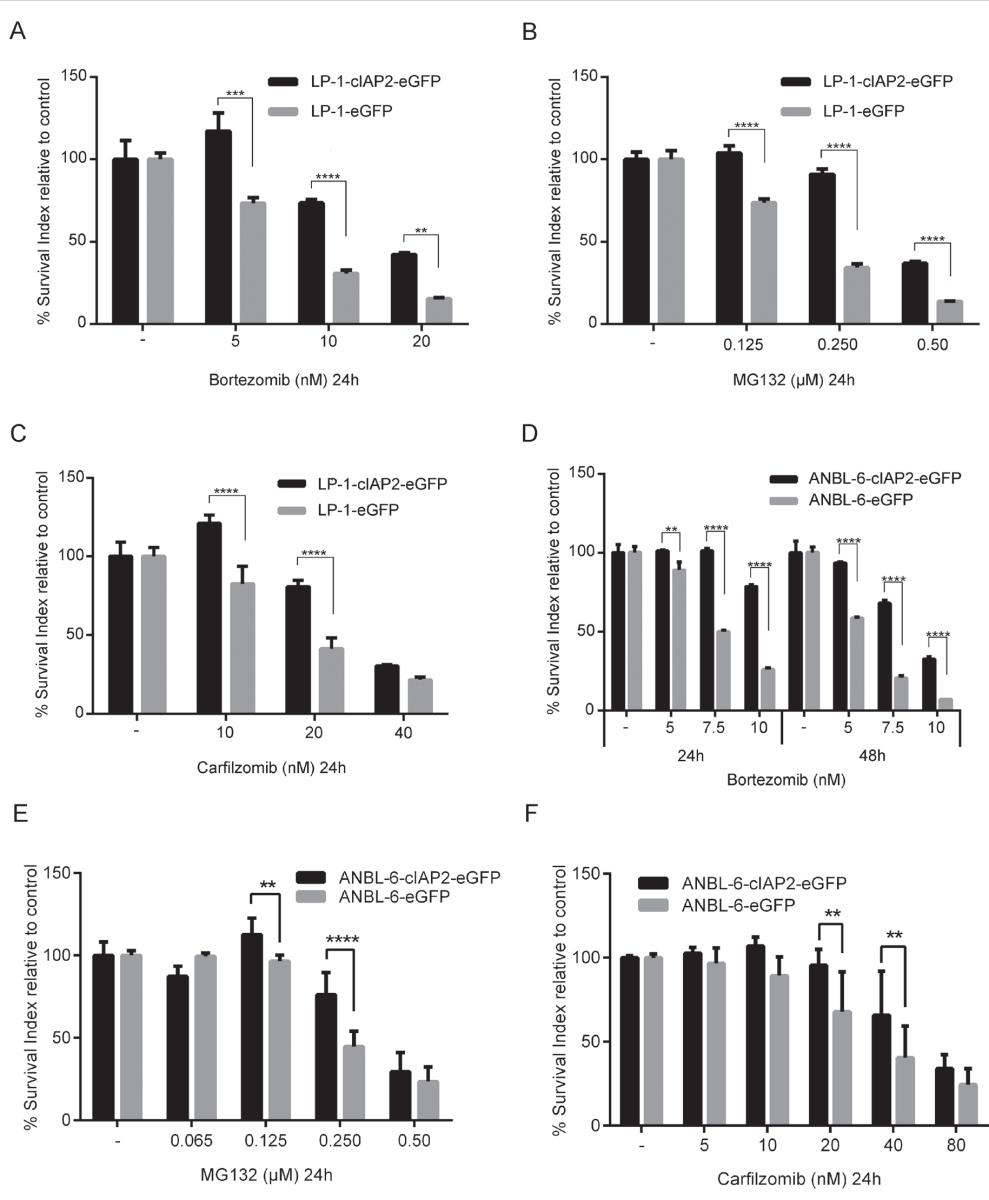
cIAP2 overexpression leads to tolerance against proteasome inhibitors in the TRAF3 mutated/deleted MM cell lines LP-1-cIAP2-eGFP and control (LP-1-eGFP) cells were incubated for 24 hours with indicated concentrations of bortezomib **A.** MG132 **B.** or carfilzomib **C.** followed by resazurin assay. ANBL-6-cIAP2-eFP and control (ANBL-6-eGFP) cells were incubated 24 and 48 hours with different concentrations of bortezomib followed by resazurin assay **D.** or 24 hours with different concentrations of MG132 **E.** or carfilzomib **F.** Three experiments were performed in triplicates and one representative is shown (A-D) whereby data are presented as mean ± SD or results are presented as mean ± SD from three biological experiments (E-F). (*indicates *p* ≤ 0.05, **p ≤ 0.01, ***p ≤ 0.001 and ****p ≤ 0.0001).

The expression of cIAP1 and cIAP2 was analyzed in a cohort of MM patients and in bone marrow plasma cells from healthy donors, MGUS and smoldering patients (Figure 1). For cIAP2 no significant differences were observed between the groups, while cIAP1 expression was increased in MM patients compared to normal and MGUS plasma cells. Furthermore, we observed that the level of cIAP2 expression was heterogeneous amongst MM patients while cIAP1 is more homogeneously expressed. The basal cIAP2 expression was similar in LP-1 and ANBL-6, as analyzed by qRT-PCR (Supplementary Figure 2). LP-1 cells overexpressing cIAP2 were generated by lentiviral transduction using pIRES2-cIAP2-eGFP vector (LP-1-cIAP2-eGFP) and pIRES2-eGFP vector (control). The increase in cIAP2 expression was verified both on mRNA and protein level (Supplementary Figure 3). To evaluate whether cIAP2 overexpression had an effect on drug resistance the cells were exposed to a panel of different drugs including the mTOR inhibitor (rapamycin), all-trans retinoic acid (atra), 17-AAG/geldanamycin (data not shown), Parthenolide, Melphalan, the histone deacetylase inhibitor suberoylanilide hydroxamic acid (SAHA), (Supplementary Figure 4) and three proteasome inhibitors. The LP-1-cIAP2-eGFP cells showed a selective tolerance to proteasome inhibitors, bortezomib (Figure 2A), MG132 (Figure 2B) or carfilzomib (Figure 2C) as compared to control.

To confirm these results we transduced the ANBL-6 cell line with the same vectors and treated them with bortezomib (Figure 2D), MG132 (Figure 2E) or carfilzomib (Figure 2F). Similarly to the results using the LP-1 cell line, we found that ANBL-6-cIAP2-eGFP cells were more tolerant to proteasome inhibitors compared to ANBL-6-eGFP (control).

### cIAP2 binds to caspases and its overexpression reduces caspase activation

**Figure 3:**
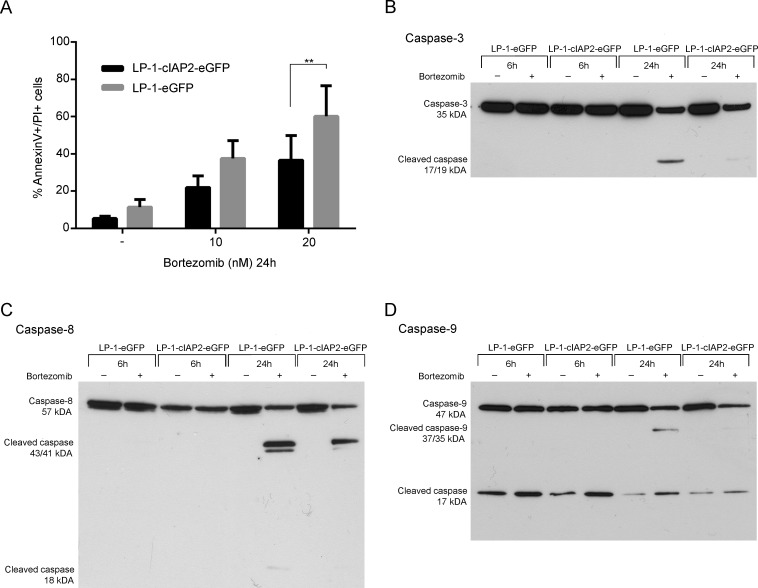
cIAP2 overexpression decreases the amount of apoptotic cells and reduces activation of caspases compared to the control **A.** LP-1-cIAP2-eGFP and control (LP-1-eGFP) cells were incubated with 10 and 20nM bortezomib for 24 hours followed by Annexin V/PI staining and flow cytometry analysis. Result is presented as mean percentage apoptotic and late apoptotic/necrotic cells ±SD (*n* = 4). (B-D) LP-1-cIAP2-eGFP and control (LP-1-eGFP) cells were incubated with 20nM bortezomib for 6 and 24 hours. Cells were harvested and protein extracts were prepared and analyzed by western blot using antibodies specific for caspase-3 **B.**, caspase-8 **C.** and caspase-9 **D.** One representative result is shown from three independent experiments. (*indicates *p* ≤ 0.05, **p ≤ 0.01, ***p ≤ 0.001 and ****p ≤ 0.0001).

We first determined the effect on apoptosis of bortezomib treatment in the LP-1-cIAP2-eGFP cells. We found a significantly lower amount of apoptotic cells in the LP-1-cIAP2-eGFP cells as compared to control (Figure 3A).

**Figure 4:**
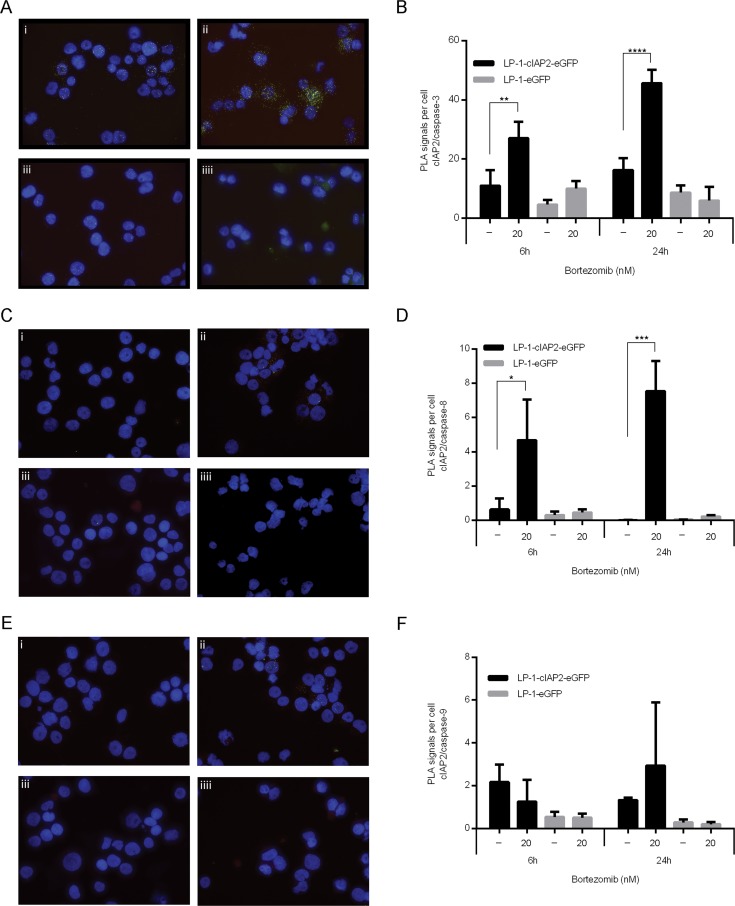
cIAP2 interacts with caspases LP-1-cIAP2-eGFP and control (LP-1-eFP) cells were incubated with 20nM bortezomib for 6 and 24 hours. After 24 hours, the interaction between cIAP2 and caspase-3 **A.**, caspase-8 **C.** or caspase-9 **E.** was assessed by *in situ* PLA (shown as green dots). i = LP-1-cIAP2-eGFP untreated, ii = LP-1-cIAP2-eGFP treated with 20nM bortezomib, iii = control (LP-1-eGFP) untreated and iiii = control (LP-1-eGFP) treated with 20nM bortezomib. One representative image out of three is shown (*n* = 2). The number of PLA signals per cell was quantified with caspase-3 **B.**, caspase-8 **D.** and caspase-9 **F.** both after 6 and 24 hours. One representative result is shown (*n* = 2) and presented as mean signals per cell ±SD. (*indicates *p* ≤ 0.05, **p ≤ 0.01, ***p ≤ 0.001 and ****p ≤ 0.0001).

Next, the effect of cIAP2 overexpression on caspase activation was examined. The results show reduced activation of caspase-3, -8 and -9 in the LP-1-cIAP2-eGFP cells as compared to control when treated with bortezomib (Figure 3B-3D). The proximity ligation assay (PLA), enables the analysis of direct protein-protein binding and was used to determine the interaction of cIAP2 to caspases at cellular level. An increased number of interactions between cIAP2 and caspases upon bortezomib treatment in the LP-1-cIAP2-eGFP was observed, illustrated as green dots (PLA signals) (Figure 4A, 4C and 4E). The quantification of the PLA signals between caspase-3 and cIAP2 demonstrated that this interaction significantly increased in LP-1-cIAP2-eGFP cells when treated with bortezomib (Figure 4Ai, 4Aii and 4B) while for the control no significant change was seen (Figure 4Aiii, 4Aiiii and 4B). The interaction between cIAP2 and caspase-8 was also significantly increased in the LP-1-cIAP2-eGFP cells when treated with bortezomib (Figure 4Ci, 4Cii and 4D) but not in the control (Figure 4Ciii, 4Ciiii and 4D). In contrast, the interactions between cIAP2 and caspase-9 was neither significantly increased in the LP-1-cIAP2-eGFP cells (Figure 4Ei, 4Eii and 4F) nor in control cells (Figure 4Eiii, 4Eiiii and 4F) upon bortezomib treatment.

### cIAP2 stabilizes the p105/p50 complex in the canonical NF-κB pathway

**Figure 5:**
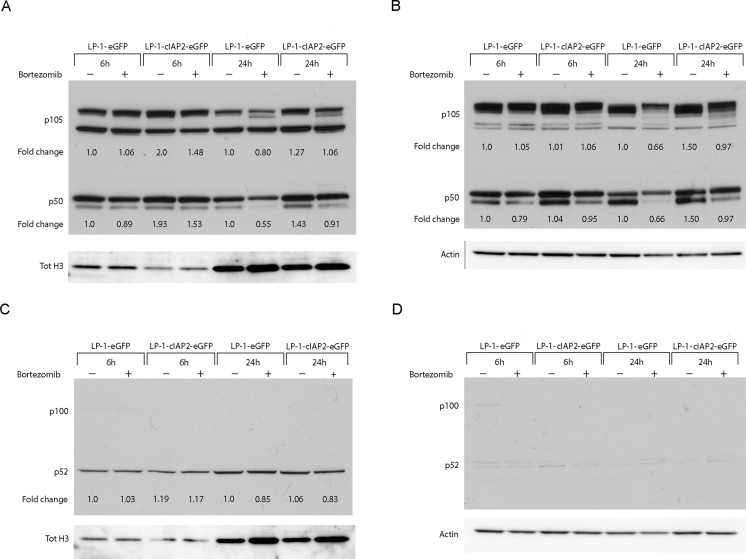
cIAP2 affects the canonical NF-κB pathway through stabilization of p105 and p50 LP-1-cIAP2-eGFP and control (LP-1-eGFP) cells were incubated with 20nM bortezomib for 6 and 24 hours. Nuclear and cytoplasmic extracts were prepared. **A.** and **C.** Western blot analysis of the nuclear extract was performed using the indicated antibodies. **B.** and **D.** Western blot analysis of the cytoplasmic extract was performed using the indicated antibodies. One representative result is shown from three independent experiments. Fold change was calculated between the protein of interest and loading control (tot H3 or actin) and then normalized to the untreated control (LP-1-eGFP).

Since cIAP2 is an important regulator of the NF-κB pathway, subcellular localization and levels of canonical (p105, p50, p65) and non-canonical pathway proteins (p100, p52) was analyzed by western blot in nuclear and cytoplasmic extracts from LP-1-cIAP2-eGFP and control cells treated with bortezomib. We found elevated levels of p105 and p50 in the nuclear (Figure 5A) and cytoplasmic (Figure 5B) fractions in the LP-1-cIAP2-eGFP cells when treated with bortezomib compared to control. No alteration or subcellular localization of the non-canonical pathway proteins, p100 and p52 was seen in the LP-1-cIAP2-eGFP or in the control cells when treated with bortezomib (Figure 5C-5D).

### The molecular mechanisms underlying the effects of cIAP2 overexpression

**Figure 6:**
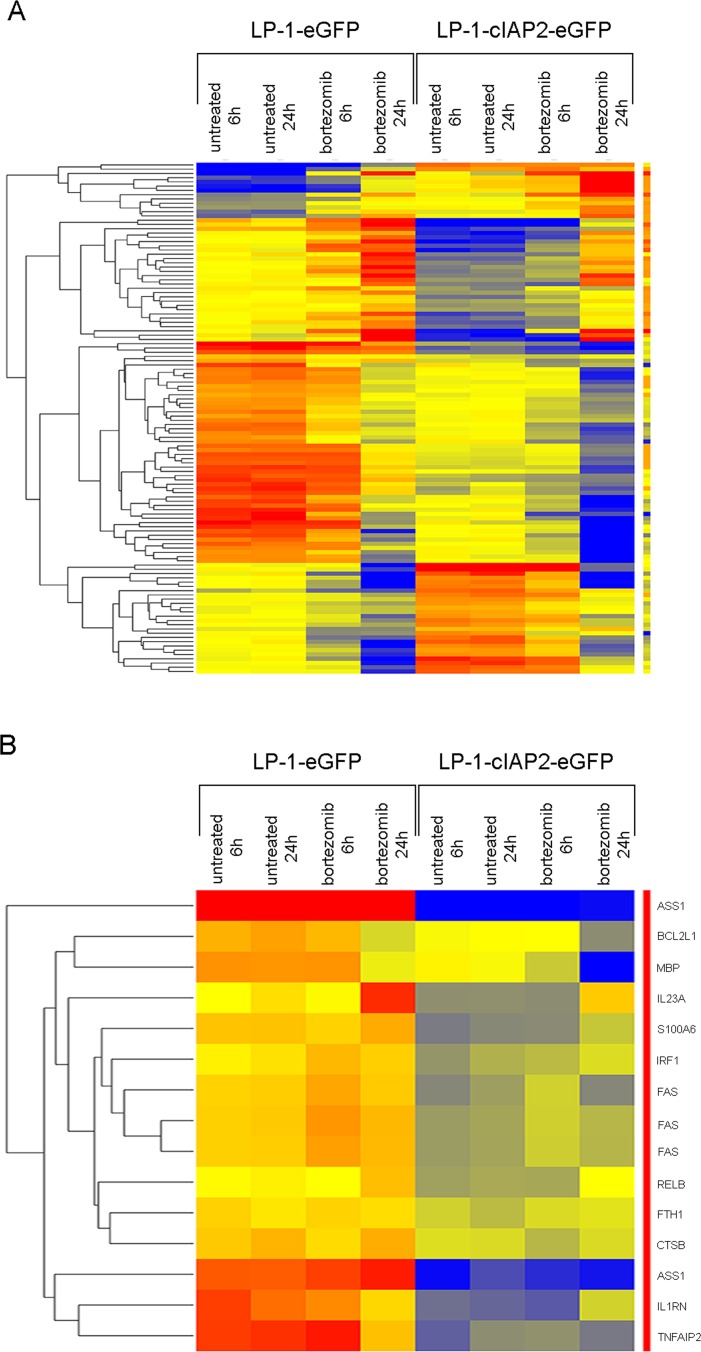
Molecular analysis of cIAP2 overexpression and bortezomib treatment **A.** Hierarchical clustering of 440 genes differentially regulated between LP-1-cIAP2-eGFP and control (LP-1-eGFP) cells. Red color is for up-regulated genes, blue color is for down-regulated genes and yellow color is for unregulated genes. **B.** Hierarchical clustering of NF-κB target genes, from the 440 gene list, that are differentially regulated between LP-1-cIAP2-eGFP and control (LP-1-eGFP) cells. Red color is for up-regulated genes, blue color is for down-regulated genes and yellow color is for unregulated genes.

To evaluate the molecular mechanisms underlying the effects of cIAP2 overexpression, a gene expression microarray was performed on 6 and 24 hours bortezomib treated LP-1-cIAP2-eGFP and control cells. The in-depth analyses revealed 1310 genes to be differentially regulated using the 3-way ANOVA and 757 genes when using the moderated t-test. In LP-1-cIAP2-eGFP versus control, 440 genes were found to be commonly regulated in both analyses. The above obtained list of 440 differentially regulated genes was then subjected to hierarchical clustering (Figure 6A) to make a direct interaction network map using the NLP (Natural Language Processing) in GeneSpring 12.6.1 (Supplementary Figure 5). Up-regulated genes that were found to have maximum interacting partners in the LP-1-cIAP2-eGFP compared to the control were IGF1, ESR2, PSAT1, TCEAL1 and CD44. In the same manner, down-regulated genes were FAS, ICAM1, CALM3, BCL2L1, FSCN1, IRF1, RUNX1, PROS1 and STAT1. From the generated 440 list we found 12 target genes of the NF-κB pathway to be down-regulated in LP-1-cIAP2-eGFP versus control (Figure 6B). Several of the NF-κB target genes were also found in the interaction map (e.g. FAS, IRF1 and BCL2L1). Other genes that were down-regulated in LP-1-cIAP2-eGFP were IL1RN, TNFAIP2, MBP, IL23A, RelB, FTH1, CTSB, S100A6 and ASS1.

### Pharmacological inhibition of IAPs leads to an increased sensitivity to bortezomib

**Figure 7:**
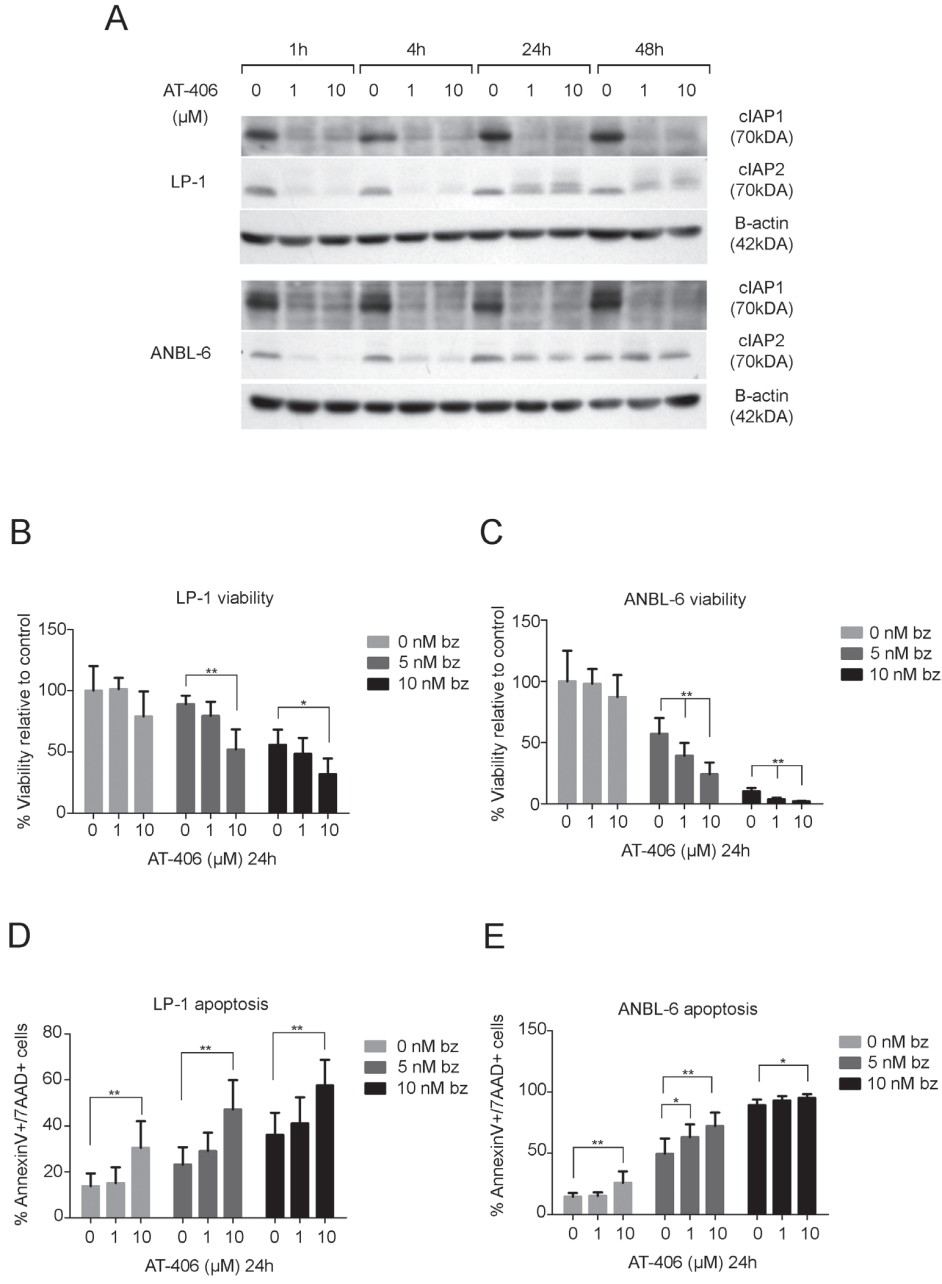
AT-406 down-regulates cIAP1 and cIAP2 and sensitizes the LP-1 and ANBL-6 cell lines to bortezomib **A.** LP-1 and ANBL-6 cells were incubated with 1 or 10μM AT-406 for 1, 4, 24 and 48 hours. Total protein lysates were analyzed for cIAP1 and cIAP2 expression by western blot. Β-actin was used as a loading control. One representative result is shown from three independent experiments. **B.**-**E.** LP-1 and ANBL-6 cell lines were pre-treated for 4 hours with AT-406 followed by 24 hours of bortezomib treatment and assessed for viability **B.**-**C.** and apoptosis with Annexin V/7AAD staining **D.**-**E.** Result is presented as mean percentage ±SD (*n* = 6). (*indicates *p* ≤ 0.05, **p ≤ 0.01).

The above results indicate that cIAP2 may constitute an important potential therapeutic target for combinatorial use with proteasome inhibitors. To investigate potential of this combination in MM, the IAP antagonist AT-406 which binds to cIAP1, cIAP2 and XIAP with high affinities was used. The LP-1 and ANBL-6 cells were pre-treated with AT-406 (1 and 10μM) for 1, 4, 24 and 48 hours, since the proteasome is needed for the degradation of cIAPs, followed by bortezomib treatment. A down-regulation of the expression of the cIAP1 and cIAP2 protein was found with the most pronounced effect after 4 hours in both cell lines (Figure 7A). Therefore, the LP-1 and ANBL-6 cells were pre-treated for 4 hours with 1 and 10μM AT-406 followed by bortezomib treatment for 24 hours. This resulted in a significant reduction in viability as compared to treatment with bortezomib alone (Figure 7B-7C). This was confirmed in the MM cell line (U266), harboring a TRAF3 mutation, and in the MM cell line (JJN3), with no TRAF3 mutation, but a NIK mutation, both cell lines dependent on the canonical pathway. The RPMI 8226 also harbors a TRAF3 mutation, although less dependent on the canonical pathway. This is also reflected by no significant effects of the combination of bortezomib and AT406 in this cell line. The OPM-2 cell line that does not harbor a TRAF3 mutation is sensitive to bortezomib but does not show enhanced apoptosis when combined with AT-406 (Supplementary Figure 6). To further confirm these results we examined the effect of the combination on apoptosis and found a significantly higher amount of apoptotic cells when combining AT-406 with either bortezomib (Figure 7D-7E) or the more novel proteasome inhibitor carfilzomib (Supplementary Figure 7) compared to single treatments.

## DISCUSSION

During the last decade the survival in MM patients has significantly improved due to the introduction of novel agents (e.g. bortezomib, thalidomide and lenalidomide) but despite these improvements, MM is still regarded incurable mainly due to the development of resistance. During disease progression drug resistant and more aggressive clones emerge which calls for the identification of new battery of targeted drugs that may be used in combination with the currently used ones. An interesting group of proteins that are often overexpressed in cancer are the members of the IAP protein family. The IAP proteins are a heterogeneous group of anti-apoptotic molecules overexpressed in several hematological malignancies [27] and associated with chemoresistance, disease progression and poor prognosis [12, 13]. Although an anti-apoptotic role for XIAP is well recognized, the function of cIAPs is still poorly understood [28]. In human MM cell lines XIAP and cIAP1 are expressed in rather similar levels, while cIAP2 is variably expressed [28]. This is in accordance with our analysis of cIAP1/cIAP2 expression in a cohort of 345 MM patients. Taken together, the IAPs could have a possible role in drug resistance and may therefore constitute a new and interesting therapeutic target in MM. In this study, we explored the possible role of cIAP2 in drug resistance. Overexpressing cIAP2 in TRAF3 deleted/mutated cell lines, LP-1 and ANBL-6, did not lead to a general drug resistance but, a decreased sensitivity to the proteasome inhibitors bortezomib, MG132 and carfilzomib.

XIAP has been described to inhibit caspase-3, -7 and -9 via its BIR2 and BIR3 domain and is therefore considered mediating the anti-apoptotic effect via this mechanism [16]. The results from the PLA studies suggest that cIAP2 indeed directly binds to caspase-3, -8 and -9. Interestingly, after treatment with bortezomib a significant increase of cIAP2 bound to caspase-3 and caspase-8 was found in the cIAP2 overexpressing cells compared to control. However, cIAPs have previously been reported to exhibit less potential to inhibit caspases due to the lack of interacting surfaces required for protease inhibition [17]. cIAPs may also obstruct apoptosis by targeting caspases for proteasomal degradation via their RING domain [29] and thus rather act primary as E3 ligases. Since a selective decrease in sensitivity to proteasome inhibitors was observed, a general caspase sequestering by the cIAP2 may not alone be a likely explanation. cIAP2 may also exert its anti-apoptotic effects via the tumor necrosis factor pathway, where they may modulate both the canonical and the non-canonical NF-κB pathway by ubiquitinating the receptor-interacting protein (RIP) and the TNF receptor-associated factor (TRAF). In the absence of cIAPs, RIP1 forms a complex with the death receptor complex and activates the extrinsic pathway and caspase-8 [30, 31]. This pathway was not the subject for examination in this study, however, we did observe an increase in active caspase-8 in the control compared to the LP-1-cIAP2-eGFP cells.

The NF-κB pathway stimulates growth and survival in MM cells [10] and the cIAPs have an important role in regulating the NF-κB signaling pathway by positive regulation of the canonical signaling and a repressive role in the non-canonical activation [27]. To determine the relative contribution of the two pathways upon cIAP2 overexpression and proteasome inhibition, we investigated the nuclear translocation of the NF-κB complexes. We demonstrated that cIAP2 overexpression leads to an increase in p105/p50 levels in nuclear and cytoplasmic extracts upon bortezomib treatment suggesting the activation of the canonical NF-κB pathway. Since the LP-1 cell line has a TRAF3 deletion/mutation and no detectable NIK, cIAP2 overexpression may not have impact on the non-canonical NF-κB pathway. We observed no alterations in the subcellular localization on p100/p52 indicating that cIAP2 overexpression did not affect the non-canonical pathway.

Gene expression analysis revealed increased expression of genes that may constitute nodes for a large number of direct interacting partners upon cIAP2 overexpression. Among these we found the IGF1 and CD44 to be up-regulated. While FAS, BCL2L1 and IRF1, all target genes of the NF-κB pathway and ICAM1, CALM3, FSCN1, RUNX1, PROS1 and STAT1 were all significantly down-regulated in the cIAP2 overexpressing cells.

NF-κB transcription factors are regulators of immune inflammatory response but are also involved in the control of apoptosis and cell proliferation in many types of cancer including MM [32]. Mutations in the NF-κB pathway may lead to overactivation of positive regulators or loss of negative regulators [9] resulting in a constitutive or overactivated NF-κB signaling leading to increased survival. In this study we found 12 target genes of NF-κB pathway to be down-regulated. To date, the complete function of all these genes are not known, however, several of them have been reported to have a tumor-suppressor role or contribute to apoptosis. ASS1 [33] and IRF1 [34] have been reported to act as tumor-suppressors. IL1RN inhibits PGE2, IL-6 production and MM cell growth [35], FTH1 is a favorable prognostic protein but is not really known to contribute to anti-tumor response [36] and the death receptor FAS induces apoptosis in a large number of cell types [37]. Gene expression analysis did not reveal regulation of the proteasome subunit β5 but interestingly we found an up-regulation of IGF-1 in the LP-1-cIAP2-eGFP cells that persisted upon treatment whereas in the control cells IGF-1 expression decreased after bortezomib treatment. IGF-1 is an important growth factor in MM and contributes to MM pathobiology. Overexpression of IGF-1 and its receptor IGF-1R correlates with disease progression and poor patient prognosis and has been linked to bortezomib resistance [8, 38-42].

Since overexpression of cIAP2 contributes to bortezomib resistance in cell lines that have TRAF3 mutation/deletion inhibition of cIAP2 by physiological or pharmacological interventions may be an attractive strategy to increase the sensitivity to bortezomib. The IAP antagonist AT-406 has been demonstrated to bind to IAPs with high affinities [43]. Due to the redundant function of the IAPs it has been shown that it is crucial to down-regulate all IAPs simultaneously to induce apoptosis [28]. Binding of AT-406 prevents binding of XIAP to caspases while binding of AT-406 to cIAP1/2 stimulates auto-ubiquitination and subsequent proteasomal degradation of cIAPs [43]. Since the proteasome is needed for the degradation of cIAPs, MM cells were pretreated with AT-406 followed with proteasome inhibition. A potent down-regulation of cIAP1 and cIAP2 were found in both cell lines already after 1 hour of treatment, cIAP1 was consistently down-regulated during all time points while cIAP2 reached baseline levels again after 24hours. In this study we could observe that AT-406 alone at a concentration of 10μM has a small but significant effect on the viability of MM cells and an enhanced sensitivity to bortezomib was found when the MM cells were pre-treated with AT-406. Currently, IAP inhibition is under extensive investigation in a wide variety of cancers. AT-406 has an *in vitro* anti-tumor effect in breast and ovarian cancer but has a minimal toxicity in normal-like human breast epithelial cells and primary human normal prostate epithelial cells [43]. This was also true for cells highly relevant to the MM microenvironment such as bone marrow stromal cells isolated from 5T33MM diseased mice, and the bone marrow endothelial cell line STR-10 (data not shown). Moreover, AT-406 is very potent inducing apoptosis in xenograft model of breast cancer and capable of complete inhibition of tumor growth [43, 44]. In preclinical xenograft models of plasmacytoma, Smac mimetics have been shown to inhibit human MM cell growth *in vivo* [45]. This model does not, however, examine the impact of the bone marrow microenvironment on tumor growth. Further studies in relevant syngeneic models of MM are necessary to understand the role of IAP antagonists within the tumor milieu in MM. AT-406 is currently in Phase I trial as a single treatment for solid tumors and lymphomas, and in trials using the combination with daunorubicin and cytarabine in acute myeloid leukemia (AML) [27]. As IAPs have a diverse and complex function in several processes including apoptosis, necroptosis and the NF-κB pathway, and MM patients show a pronounced genetic heterogeneity as well as in drug response, further studies are needed to elucidate the function of IAPs in MM. From our current study, we conclude that in TRAF3 mutated MM cells, cIAP2 expression is an important factor in resistance to proteasome inhibition. This resistance is caused by a decrease of cleaved caspases upon treatment, activation of the canonical NF-κB pathway, and dysregulation of genes acting as direct interaction hubs, including down-regulated NF-κB target genes with known anti-tumor activity. Furthermore, approximately 20% of the MM patients harbor genetic lesions in genes of the NF-κB pathway leading to uncontrolled NF-κB activation, loss of functional TRAF3 being the most common gene deleted/mutated [9, 24]. We show that inhibition of IAPs could increase the sensitivity to bortezomib, thus suggesting that a combination of IAP antagonists with bortezomib would be beneficial for MM patients harboring TRAF3 mutations leading to hyperactivation of the NF-κB pathway and more dependency on the canonical pathway.

## MATERIALS AND METHODS

### Analysis of cIAP1 and cIAP2 gene expression in MM patients

Gene expression levels were analyzed in publically available datasets of pre-treatment bone marrow aspirates from 414 MM patients and bone marrow plasma cells from healthy donors (*n* = 22), MGUS (*n* = 44), and Smoldering Myeloma (*n* = 12) from the University of Arkansas for Medical Sciences (Little Rock, USA) [46]. These data can be accessed at the online Gene Expression Omnibus (GSE4581 and GSE5900). Normalization of gene expression data was performed using the MAS5 algorithm and analyzed by the bioinformatics Platform Genomicscape (http://genomicscape.com/) [47]

### Cell lines

Human MM cell lines LP-1 [48] (DSMZ) and ANBL-6 (a kind gift from Prof Jelinek), all authenticated by STR analysis, were maintained in RPMI-1640 (Lonza, Basel, Switzerland) supplemented with 10% fetal bovine serum (Biochrom AG, Berlin, Germany), glutamine (2mM) and antibiotics (penicillin 100U/mL and streptomycin 50g/mL) (Lonza) at 37°C in a humidified 5% CO2 in-air atmosphere. The ANBL-6 cell line was supplemented with 2ng/ml IL-6 (R&D systems, Abingdon, UK).

### Lentiviral production and stable transduction

HEK293T cells were co-transfected with the pMD.G envelope plasmid, the pCMV packing plasmid and the transfer plasmid pIRES2 expressing cIAP2 and eGFP or only eGFP (control). Lentiviruses were produced using the lipofectamin protocol according to manufacturer's instructions (Sigma-Aldrich, St.Louis, USA). After 48 and 72 hours the supernatant containing the lentiviruses was harvested and concentrated by ultracentrifugation. The eGFP expression of transduced cells was determined using the FACSCalibur flow cytometer (BD Biosciences, Franklin Lakes, NJ, USA) and cells were sorted using the AriaIII (BD Biosciences). FACSDiva software was used for analysis.

### Reagents

Bortezomib (Selleckchem, Munich, Germany), MG132, all-trans retinoic acid (atra), Parthenolide, Melphalan (Sigma-Aldrich, St. Louis MO, USA), AT-406 (MedKoo Biosciences, Chapel Hill, NC, USA), Rapamycin (Merck Biosciences, Darmstadt, Germany), 17-AAG/geldanamycin and suberoylanilide hydroxamic acid (SAHA) (Biovision, Milpitas, USA) were dissolved in dimethyl sulfoxide (DMSO; final concentration <0.01%) and aliquots were stored at −20°C.

### RNA extraction, cDNA synthesis and quantitative real-time PCR

Total RNA was extracted using TRIzol (Invitrogen, Carlsbad, CA, USA) and reverse transcription using random primers (Invitrogen) was performed on 2μg total RNA using SuperScript^™^ III reverse transcriptase (Invitrogen) both according to manufacturer's instructions. cDNA was analyzed by quantitative real-time RT-PCR using Platinum® SYBR®Green pPCR Supermix UDG with Rox (Invitrogen) and 0.3mM of cIAP2 forward primer 5′-gtg tta gac tta ctc aat gca gaa g-3′ and 0.3mM of reverse primer 5′-cca gga ttg gaa tta cac aag tc-3′. The run and analysis were performed using Mx3005P (Stratagene).

### Drug sensitivity of transduced cells

The viability of the transduced cells upon treatment was assessed with the Resazurin assay using AlamarBlue (Sigma-Aldrich) as previous described [49].

Apoptosis was quantified by staining with annexinV-Alexa Fluor 647 and Propidium Iodide (PI) (Invitrogen). Cells were analyzed with FACSCalibur flow cytometer (BD Biosciences) using FACSDiva software according to manufacturer's instructions.

### Combination effect of IAP inhibitor AT-406 with bortezomib

LP-1(wt) and ANBL-6(wt) cells were treated for 4 hours with 1 or 10μM of AT-406 followed by bortezomib treatment (5 or 10nM). After 24 hours the viability was measured with the CellTiter-Glo Luminiscent Viability assay (Promega, Leiden, The Netherlands) according to manufacturer's instructions. The relative amount of viable cells was expressed as percentage of untreated cells.

Apoptosis was measured with Annexin V-FITC and 7-AAD (BD Biosciences) followed by flow cytometric analysis (FACS Canto and Diva software, BD Biosciences) according to manufacturer's instructions.

### Western blot analysis

Cells were harvested, lysed, and protein extracts were blotted as previously described [50]. Primary antibodies were used against caspase-3, caspase-8, caspase-9, cIAP2 (Cell Signaling, Leiden, The Netherlands), cIAP1 (R&D systems) p105, p50, p100, p52 and β-actin (Santa Cruz, Heidelberg, Germany). β-actin was used as a loading control.

### Proximity ligation assay

Cells were treated with bortezomib (20nM) for 6 and 24 hours. Approximately, 50.000-100.000 cells were spun on glass slides, fixed in 4% PFA and permeabilized in EtOH 70% at 4°C. Samples were assessed as previously described [51]. The short and the long circularization oligonucleotide and the fluorophore labelled oligonucleotide are listed in (Supplementary Table 1). Raw images were used for quantification of signals per cell in CellProfiler version one (www.cellprofiler.org).

### Gene expression array

Cells were treated with 10nM bortezomib for 6 and 24 hours. Total RNA isolation was performed using illustra RNAspin mini RNA isolation kit (GE Healthcare, Uppsala, Sweden) according to manufacturer's protocol. Gene expression microarrays were performed using Agilent Sure Print G3 Human gene expression 8×60K v2 microarray. The labeling was done according to manufacturer's protocol. Two biological replicates were used for microarray analysis. In the LP-1-cIAP2-eGFP cells, a lower expression of cIAP2 (BIRC3) was found compared to the control in the array. This is due to the design of the probe on the array to the 3 prime UTR detecting only the endogenous cIAP2. RT-qPCR analysis was used to verify that the LP-cIAP2-eGFP cells overexpress the cIAP2 mRNA as compared to control (Supplementary Figure 1).http://www.ncbi.nlm.nih.gov/geo/query/acc.cgi?token=kvqjyigmpxcpvid&acc=GSE63520

### Statistical and clustering analysis

The arrays were Quantile normalized using GeneSpring12.6.1. 3-way ANOVA with a FDR correction using Benjamini-Hochberg was used to find differentially regulated genes in the untreated LP-1-cIAP2-eGFP compared to untreated control using three parameters i.e. time, treatment and cIAP2 expression. The *p*-value < 0.001 and a fold change >2-fold were used as a cutoff for selecting differentially regulated genes. A moderated t-test was used to evaluate the effect on gene expression as a result of cIAP2 overexpression with a p-value of 0.005 and >2-fold cutoff. The hierarchical clustering was done for >2-fold differentially regulated genes using Euclidian distance and average linking rule. Further bioinformatics analysis was performed using genes selected to be differently regulated in the LP-1-cIAP2-eGFP versus control. To compare cIAP expression levels between normal, MGUS, smoldering and MM cells, an unpaired student t-test was used. For all other experiments 2-way ANOVA or Mann-Whitney U-test were used to calculate the significance. A *p*-value < 0.05 was considered as significant.

## SUPPLEMENTARY FIGURES AND TABLE


