# Supplementary material for: Increased resistance to proteasome inhibitors in multiple myeloma mediated by cIAP2 - implications for a combinatorial treatment
Source: Oncotarget. 2015 May 14;6(24):20621–35. doi: 10.18632/oncotarget.4139 (PMC4653030; doi:10.18632/oncotarget.4139)
Supplement: Supplementary file 1 [file oncotarget-06-20621-s001.pdf]

Increased resistance to proteasome inhibitors in multiple myeloma mediated by cIAP2 - implications for a combinatorial treatment

Supplementary Material

Supplemental table

Table S1: Oligonucleotides used for *in situ* PLA

Oligonucleotide	Vendor	DNA sequence
Short circularization oligonucleotide	Integrated DNA technology	5' – GTTCTGTCATATTTAAGCGTCTTAA - 3'*
Long circularization oligonucleotide	Integrated DNA technology	5' - CTATTAGCGTCCAGTGAATGCGAGTCCGTCTAAGAGAGTAGTACAGCAGCCGTCAAGAGTGTCTA - 3'*
Cy3 labelled oligonucleotide	Integrated DNA technology	5' -CAGTGAATGCGAGTCCGTCTZZZZ - 3'**

* The oligonucleotide is phosphorylated on the 5´end.

**Z represents 2´O methyl-RNA

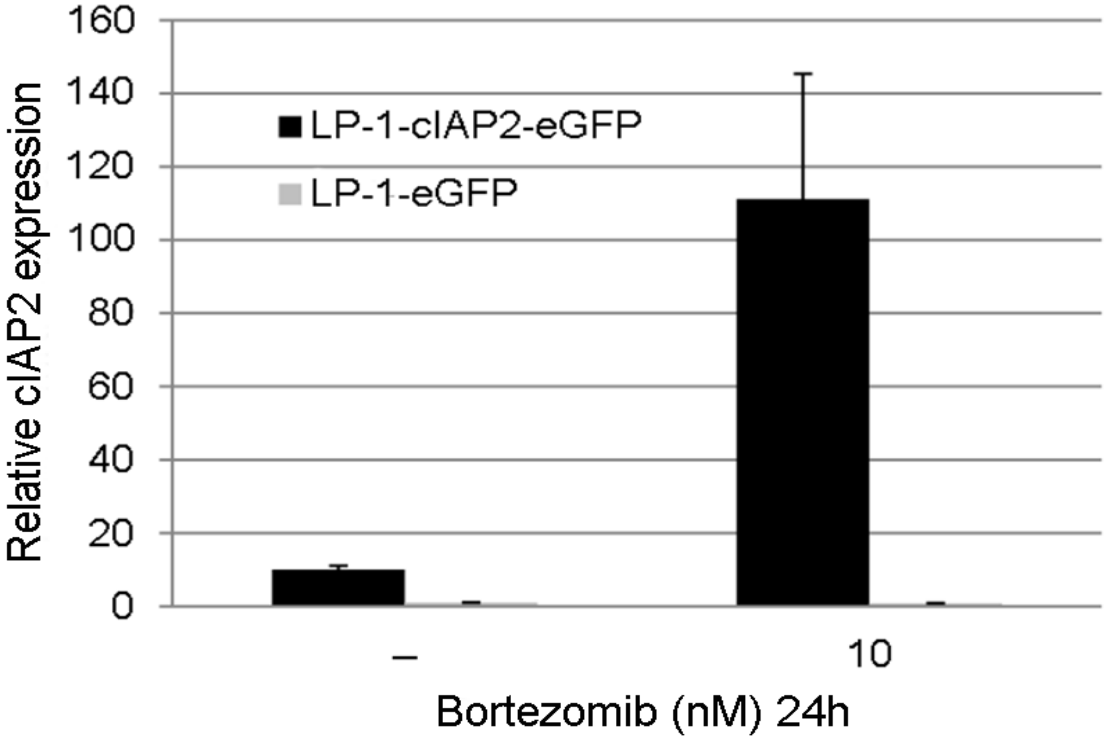

Supplementary figure 1. **cIAP2 expression in LP-1-cIAP2-eGFP and LP-1-eGFP cells.** The cIAP2 expression in LP-1-cIAP2-eGFP and LP-1-eGFP cells, with and without bortezomib treatment, were assessed with qRT-PCR. Two experiments were performed in triplicates and one representative is shown as relative expression, normalized to GAPDH mean ± SD (n=2).

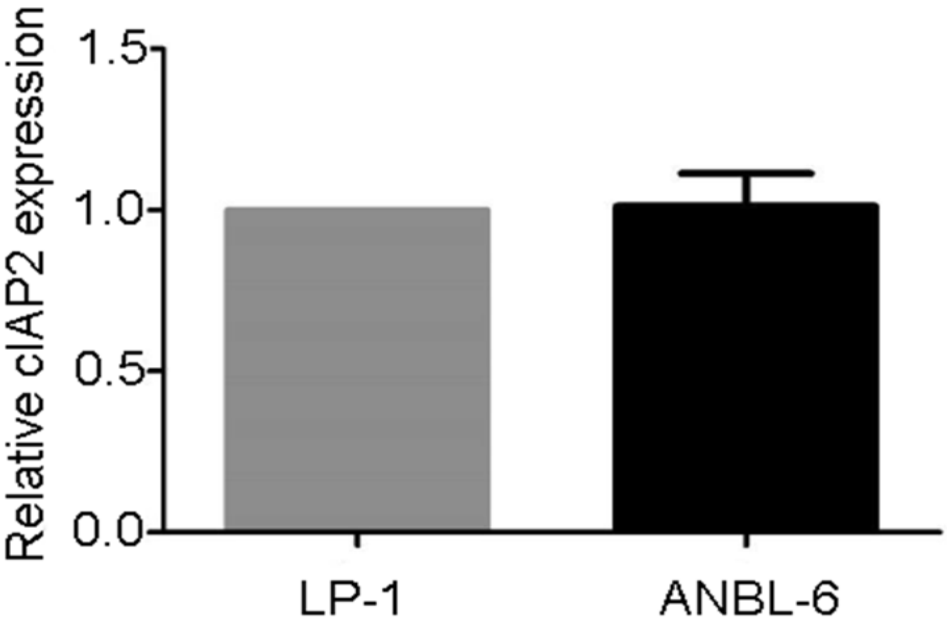

Supplementary figure 2. **LP-1 and ANBL-6 have similar cIAP2 expression.** The cIAP2 expression in the LP-1 and ANBL-6 cell line was assessed with qRT-PCR. Results are shown as relative expression, normalized to GAPDH mean ± SD (n=3).

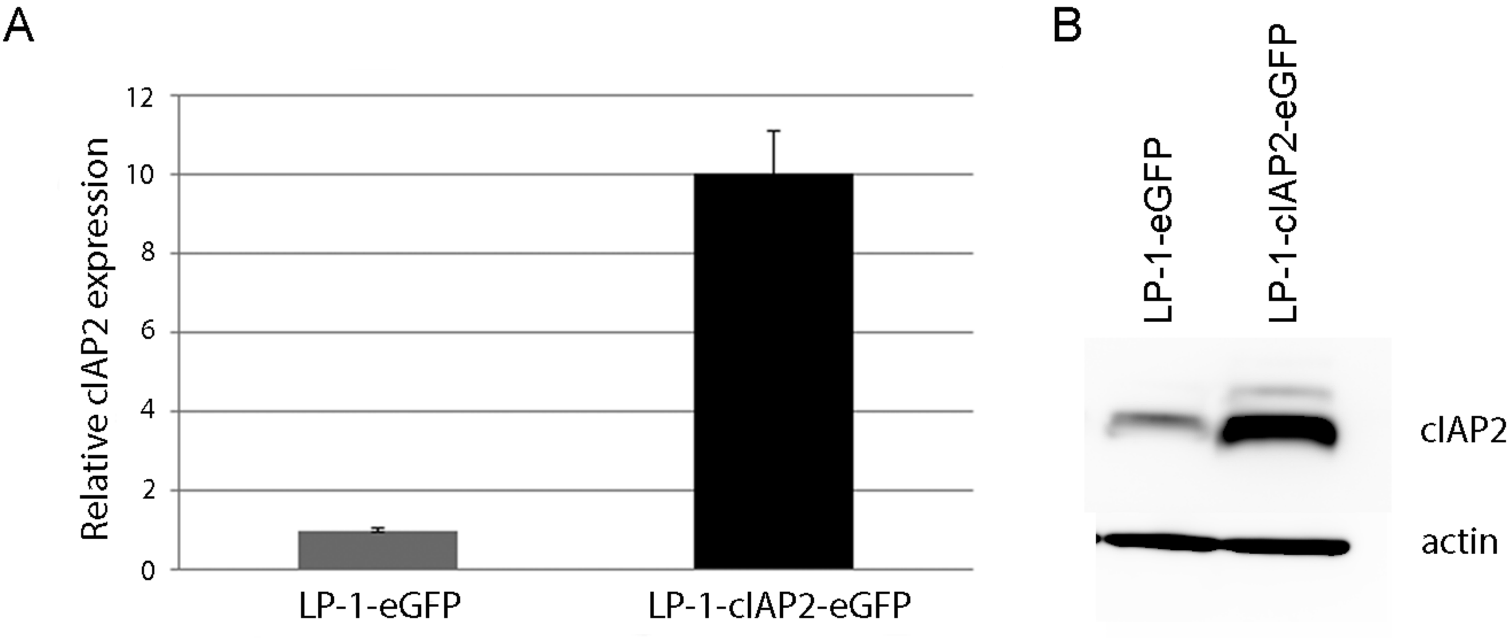

Supplementary figure 3. **Quantification of mRNA and protein levels in LP-1-cIAP2-eGFP and LP-1-eGFP cells.** The cIAP2 expression was evaluated after the lenitviral transduction. The cIAP2 mRNA expression (A) in LP-1-cIAP2-eGFP and LP-1-eGFP cells was assessed with qRT-PCR. Three experiments were performed in triplicates and one representative is shown as relative expression, normalized to GAPDH mean ± SD (n=3). (B) The protein level was analyzed using western blot. One representative result is shown from three independent experiments.

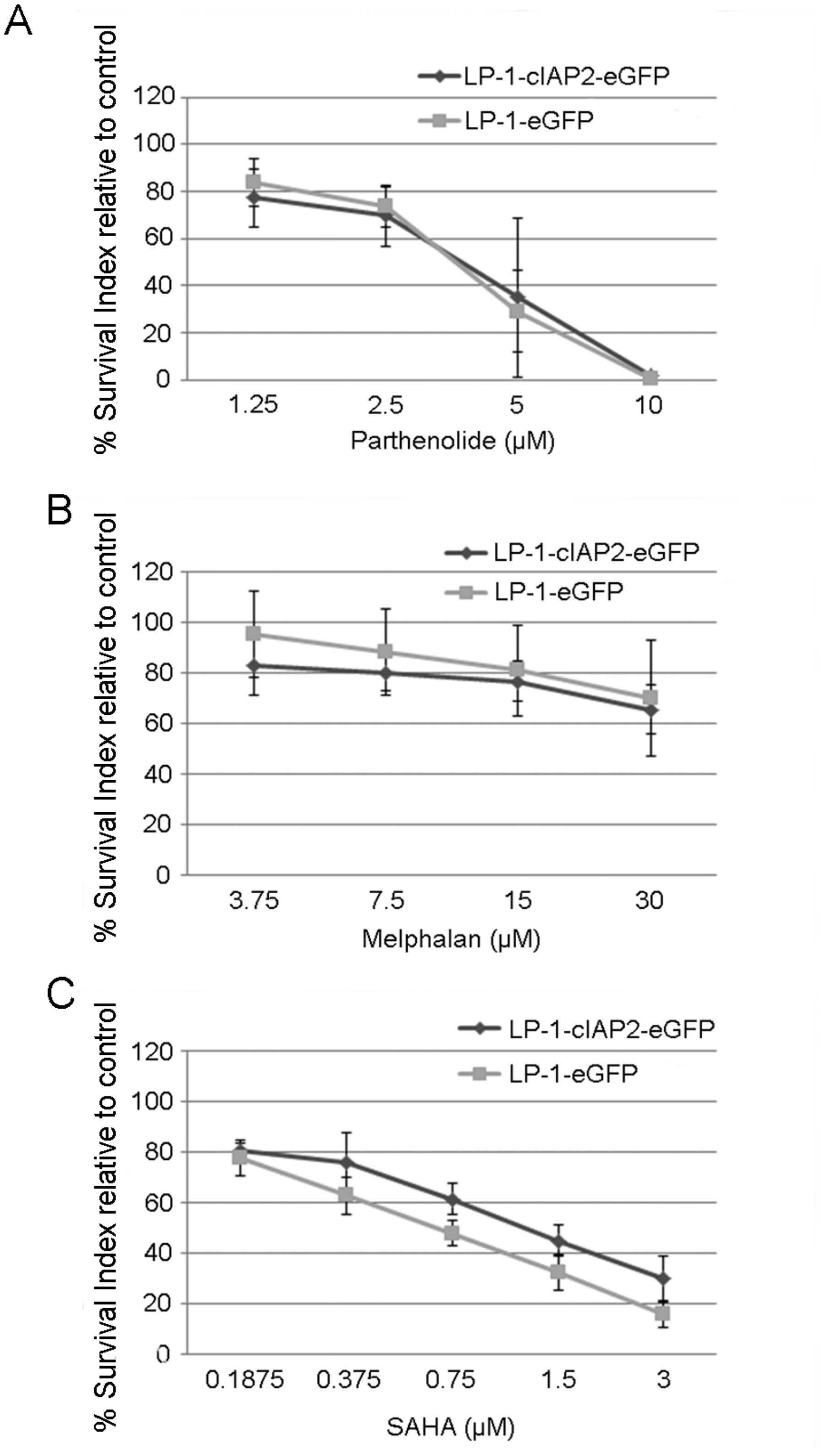

Supplemental figure 4. **Effect of cIAP2 overexpression when treated with different drugs.** LP-1-cIAP2-eGFP and the control (LP-1-eGFP) cells were incubated with different concentrations of (A)Parthenolide, (B) Melphalan and (C) SAHA for 72 hours followed by resazurin assay. Result is presented as mean percentage relative to untreated cells ±SD (n=3).

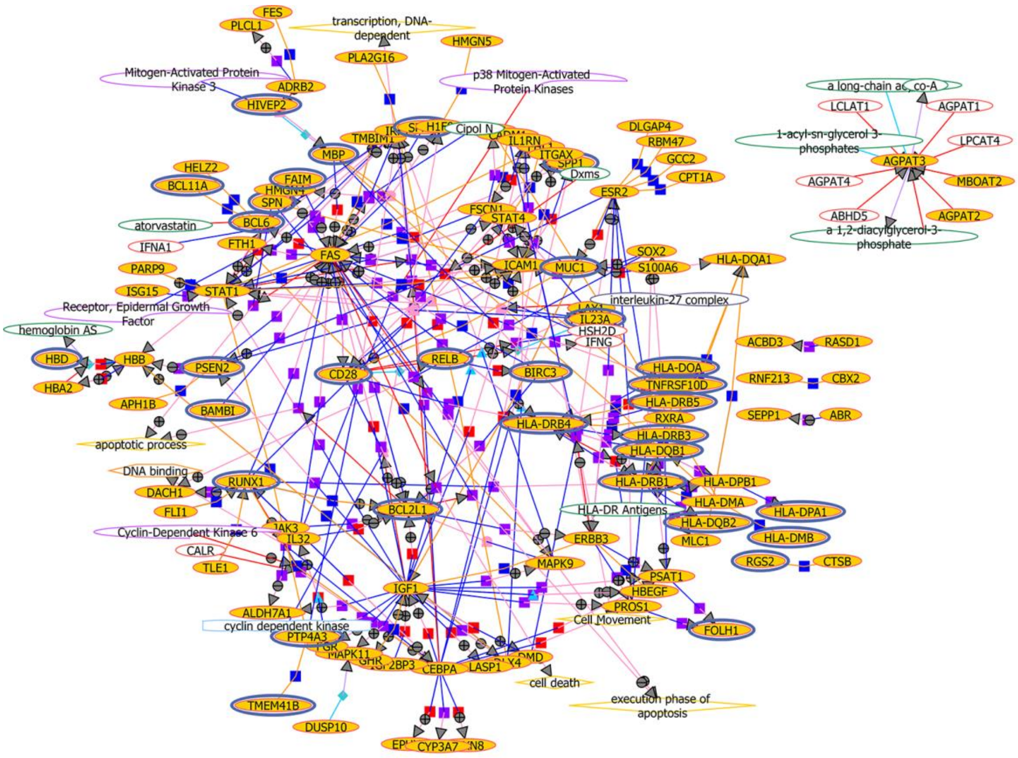

Supplementary figure 5. **Interaction network of the regulated genes.** The interaction network of the 440 differentially regulated genes between LP-1-cIAP2-eGFP and control (LP-1-eGFP) cells. The connection lines are the direct connection between the genes. Genes are indicated in the circles and the genes with several lines are having the most interacting partners.

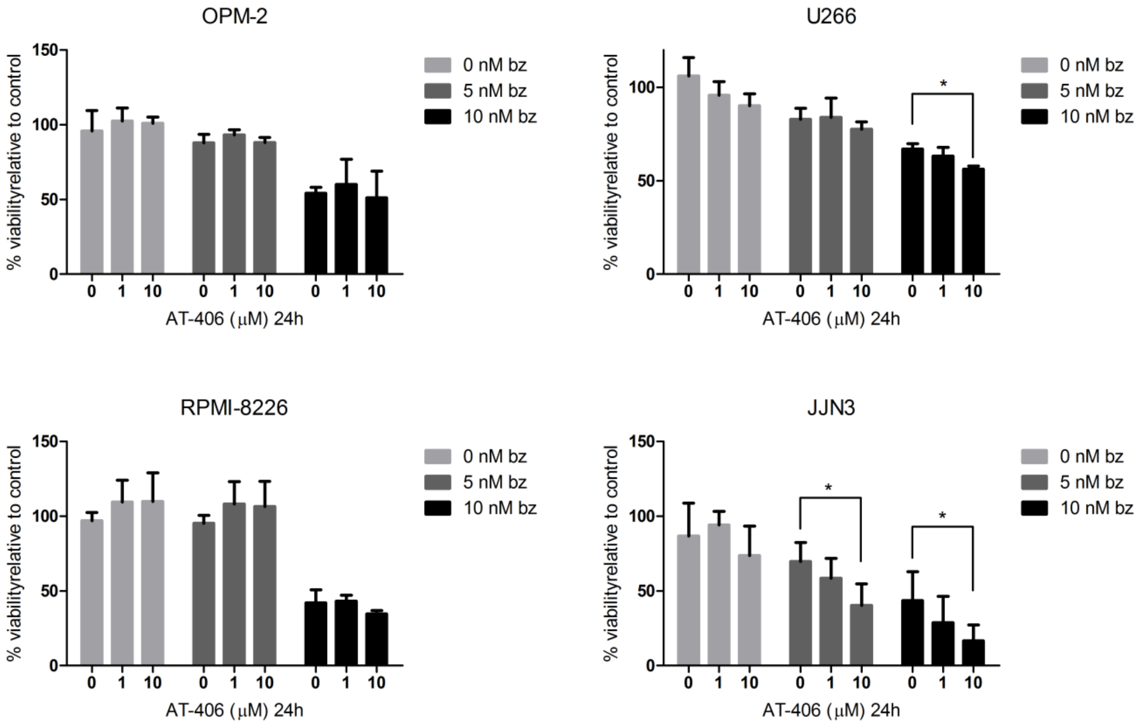

Supplementary figure 6. **Effects on viability when combining AT-406 with bortezomib in several MM cell lines.** OPM-2, U266, RPMI-8226 and JJN3 cell lines were pre-treated for 4 hours with AT-406 followed by 24 hours of bortezomib treatment and assessed for viability. Result is presented as mean percentage ±SD (n=3). (*indicates p≤0.05).

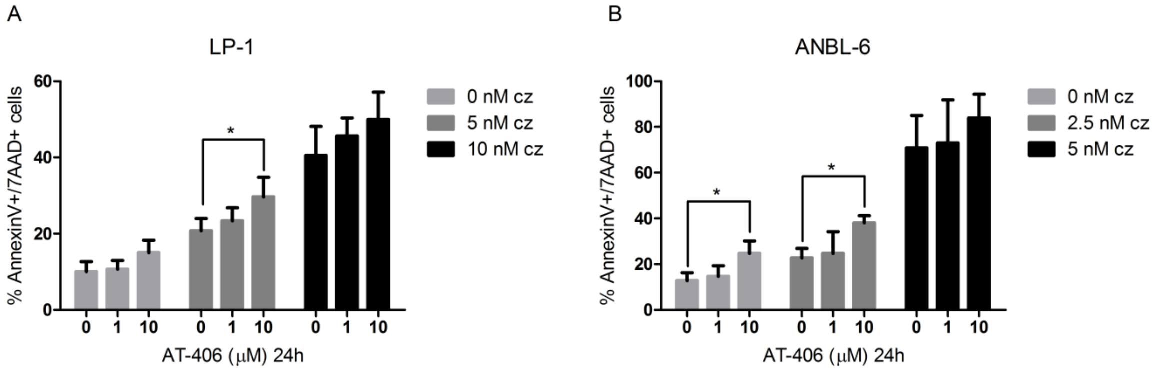

Supplementary figure 7. **Combining AT-406 with carfilzomib increased the amount of apoptotic cells in LP-1 and ANBL-6 cell lines**. LP-1 (A) and ANBL-6 (B) cell lines were pretreated for 4 hours with AT-406 followed by 24 hours of carfilzomib (cz) treatment and assessed for apoptosis with Annexin V/7AAD staining. Result is presented as mean percentage ±SD (n=3). (*indicates $p \leq 0.05$).
